# Detailed Long-Term Follow-Up of Patients Who Relapsed After the Nordic Mantle Cell Lymphoma Trials: MCL2 and MCL3

**DOI:** 10.1097/HS9.0000000000000510

**Published:** 2020-12-21

**Authors:** Christian Winther Eskelund, Kostas Dimopoulos, Arne Kolstad, Ingrid Glimelius, Riikka Räty, Lise Mette Rahbek Gjerdrum, Kristina Sonnevi, Pär Josefsson, Herman Nilsson-Ehle, Hans H. N. Bentzen, Unn Merete Fagerli, Outi Kuittinen, Jacob Haaber, Carsten Utoft Niemann, Lone Bredo Pedersen, Maria Torp Larsen, Christian Hartmann Geisler, Martin Hutchings, Mats Jerkeman, Kirsten Grønbæk

**Affiliations:** 1 Department of Haematology, Rigshospitalet, Copenhagen, Denmark; 2 Biotech Research & Innovation Centre BRIC, University of Copenhagen, Denmark; 3 The Danish Stem Cell Center (Danstem), University of Copenhagen, Denmark; 4 Department of Oncology, Oslo University Hospital, Oslo, Norway; 5 Unit of Experimental and Clinical Oncology, Department of Immunology, Genetics and Pathology, Uppsala University, Sweden; 6 Department of Haematology, Helsinki University Hospital, Helsinki, Finland; 7 Department of Pathology, Zealand University Hospital, Roskilde, Denmark; 8 Department of Clinical Medicine, University of Copenhagen, Denmark; 9 Department of Haematology, Karolinska University Hospital, Stockholm, Sweden; 10 Department of Haematology, Herlev Hospital, Herlev, Denmark; 11 Department of Haematology, Sahlgrenska University Hospital, Göteborg, Sweden; 12 Department of Haematology, Aarhus University Hospital, Aarhus, Denmark; 13 Department of Oncology, St. Olav’s Hospital, Trondheim, Norway; 14 Institute of Clinical and Molecular Medicine (IKOM), Norwegian University of Science and Technology (NTNTU), Trondheim, Norway; 15 Department of Oncology, Kuopio University Hospital, Kuopio, Finland; 16 Department of Haematology, Odense University Hospital, Odense, Denmark; 17 Department of Oncology, Skåne University Hospital, Lund, Sweden.

## Abstract

Mantle cell lymphoma (MCL) is an incurable disease with a highly variable clinical course. The prognosis after relapse is generally poor, and no standard of care exists. We investigated the postrelapse outcomes of 149 patients who were initially treated in the Nordic Lymphoma Group trials, MCL2 or MCL3, both representing intensive cytarabine-containing frontline regimens including autologous stem cell transplant. Patients with progression of disease before 24 months (POD24, n = 51, 34%) displayed a median overall survival of 6.6 months compared with 46 months for patients with later POD (n = 98, 66%; *P* < 0.001). MCL international prognostic index, cell proliferation marker, blastoid morphology, and *TP53* mutations showed independent prognostic value irrespective of POD24, and in a combined, exploratory risk score, patients with 0, 1, 2-3, or 4-5 high-risk markers, respectively, displayed a 5-year overall survival of 62%, 39%, 31%, and 0%. By a comparison of median progression-free survival of the different salvage therapies in the relapse setting, bendamustine-rituximab was superior to all other combination chemotherapy regimens; however, it was also associated with longer responses to last line of therapy. Collectively, we confirm the prognostic impact of POD24 and highlight the relevance of other biomarkers, and we emphasize the importance of novel therapies for patients with high-risk features at first POD.

## Introduction

The prognosis for younger patients with mantle cell lymphoma (MCL) has improved in the recent decades due to intensified frontline regimens and rituximab maintenance.^1-4^ The Nordic Lymphoma Group conducted 2 trials of intensive cytarabine-containing frontline regimens and autologous stem cell transplantation (ASCT), the MCL2 and MCL3 trials,^1,5^ of which the long-term follow-up of MCL2 showed a median overall survival (OS) exceeding 12 years.^6^ However, relapses occurred gradually throughout the entire follow-up period, even beyond 10 years of remission, suggesting that MCL is incurable by chemoimmunotherapy. At time of relapse, no standard of care exists, and the prognosis is poor despite the increasing use of novel agents such as ibrutinib.^7-9^ In cohorts of younger patients treated by intensive frontline regimens, Visco et al^8^ showed a notable prognostic impact of time to progression of disease (POD) with a cut-off at 24 months from start of first treatment. Similar results were presented by Kumar et al,^7^ although in a more heterogeneous cohort and with a cut-off of 12 months. However, information on regimens used at the time of POD in these studies was limited.

Here, we present the results of a multicentre, retrospective cohort study of patients who relapsed or progressed after initial treatment in the 2 prospective Nordic Lymphoma Group trials, MCL2 and MCL3, both representing current standard of care regimens for younger patients with newly diagnosed MCL.

## Methods

### Patients

**Figure 1.**
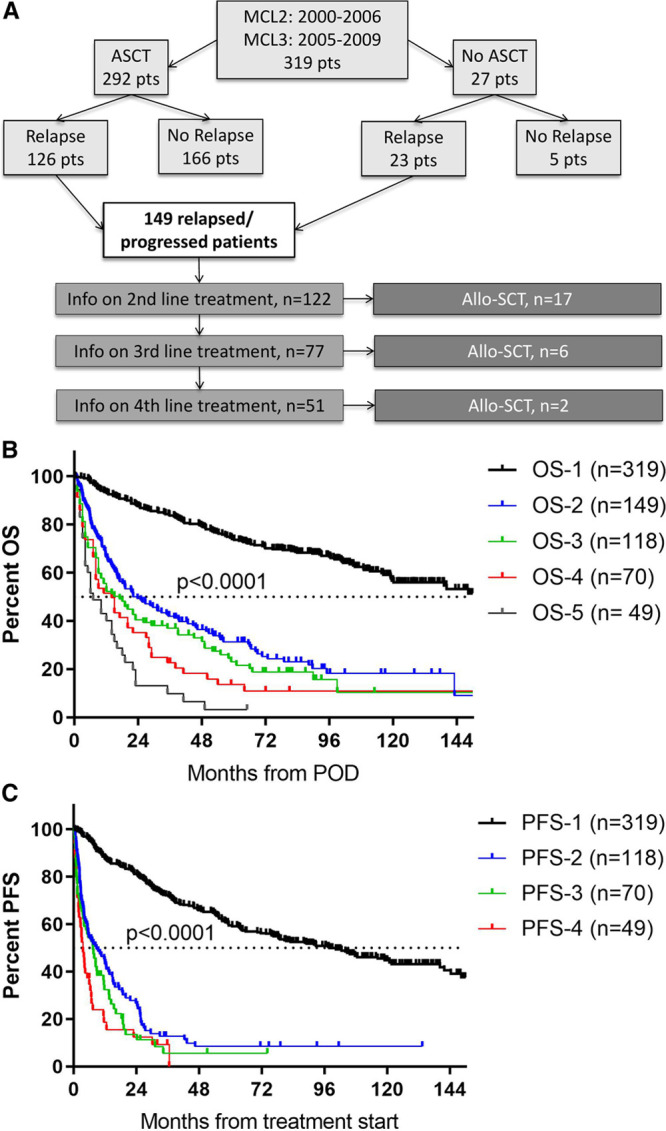
**Overview and outcome of patients from the MCL2 and MCL3 trials after POD.** (A) Flow chart showing the study cohort. (B) OS and (C) PFS in relation to line of therapy measured from time of disease progression and time of treatment start, respectively (In [A], total number of patients is included; in [B], all patients with known POD and death dates; and in [C], all patients with known dates of treatment start and of next POD if any). AlloSCT = allogeneic stem cell transplantation, ASCT = autologous stem cell transplantation, OS = overall survival, PFS = progression-free survival, POD = progression of disease.

We conducted a retrospective cohort study of all 149 patients with POD after initial treatment in the Nordic MCL trials, MCL2 and MCL3 (Figure 1A). Primary treatment of patients has been described elsewhere.^1,5^ Briefly, 319 patients younger than 66 years of age received an induction consisting of rituximab and 6 alternating courses of maxi-CHOP (cyclophosphamide, doxorubicin, vincristine, and prednisolone) and high-dose cytarabine followed by stem cell harvest and consolidation with high-dose chemotherapy, BEAM/C (carmustine, etoposide, Ara-C, and melphalan/cyclophosphamide). During follow-up, patients with minimal residual disease markers (n = 183) who experienced molecular relapses with no concomitant clinical relapse were treated pre-emptively with 4 cycles of rituximab. Only clinical relapses are included in this study.

Information on later lines of therapy was collected by contact to the local treating physician with March 2019 being the latest follow-up date. Of all 149 patients with POD, information on second, third, and fourth-line therapy was available for 122, 77, and 52, respectively (Figure 1A). Of note, complete information on treatment dates and follow-up was not available for all, and hence, the numbers do not add up for all the subsequent response and outcome analyses.

MCL international prognostic index (MIPI) was calculated according to Hoster et al.^10^ Mutations of *TP53* were analyzed by next-generation sequencing of diagnostic bone marrow or lymph node samples according to the previous study by the Nordic MCL group.^11^

### Statistics

Response evaluations were performed by the treating physician according to local guidelines. OS was measured from time of relapse to death of any cause. Progression-free survival (PFS) was measured from start of salvage therapy to next documented POD or death of any cause, and patients were censored at time of allogeneic stem cell transplantation (AlloSCT). Both OS and PFS were estimated by the Kaplan-Meier method, and comparison of covariables was done by the log-rank test. Time to progression (TTP) was measured from start of last line of therapy to POD. To evaluate the prognostic impact of TTP following frontline treatment, we used TTP in a separate Cox regression model and then plotted the predicted hazard ratios using a cubic spline model.

For multivariable comparisons, Cox regression was used. MIPI score and cell proliferation marker (Ki67) were included as continuous variables, and blastoid morphology and *TP53*-mutations as bimodal variables. Each variable was adjusted separately for the bimodal variable of early relapse (POD before 24 months [POD24]). Chi-square and Fisher exact test were used to compare categorical variables, as appropriate, and Spearman correlation was used to evaluate the relationship between continuous variables. All *P* values were 2-tailed, and statistical significance was defined as *P* < 0.05. Statistical analyses were performed in SPSS 22.0 for Windows, GraphPad Prism 7.02 for Windows, and R (version 3.5.2).

## Results

### Patient characteristics

Of 319 patients included in the 2 prospective trials, MCL2 and MCL3, for patients younger than 66 years of age with newly diagnosed MCL from 2000 to 2009,^1,5^ a total of 149 patients had POD between April 2001 and March 2019 (Figure 1A). Median age at relapse was 61 (interquartile range [IQR] 57-65) and 78% were males. Of 118 patients with available information, 7 (6%) had central nervous system involvement at POD (Table 1). MIPI status was available for 82 patients with 38 (46%) being low risk, 19 (23%) intermediate risk, and 25 (30%) high risk. There was a significant correlation of MIPI measured at diagnosis and at time of POD (ρ = 0.36 [95% CI = 0.15-0.54], *P* = 0.0008, Spearman’s correlation; Supplementary Figure 1, http://links.lww.com/HS/A121). Seventeen (40%) had Ki67 below 30% and 26 (60%) above 30% at time of POD. Like MIPI, Ki67 expression correlated significantly between diagnosis and time of POD (ρ = 0.67 [95% CI = 0.42-0.83], *P* < 0.001, Spearman’s correlation; Supplementary Figure 2, http://links.lww.com/HS/A121).

**Table 1:** Patient Characteristics.

	All Patients		Early POD, <24 mo		Late POD, ≥24 mo		*P*
	n	%	n	%	n	%	
	149		51	34	98	66	
Sex							
Female	32	21	9	18	23	23	0.41
Male	117	79	42	82	75	77	
Trial							
MCL2	78	52	26	51	52	53	0.81
MCL3	71	48	25	49	46	47	
ASCT in front line							
Yes	126	85	35	69	91	93	<0.001
No	23	15	16	31	7	7	
At first POD:							
Median age	61 (IQR 57-65)		61 (IQR 55-64)		61 (IQR 57-67)		0.14
CNS involvement (n = 118)							
No	111	94	37	88	74	97	0.10
Yes	7	6	5	12	2	3	
MIPI (n = 82)							
LR	38	46	9	41	29	48	0.17
IR	19	23	3	14	16	27	
HR	25	30	10	45	15	25	
Ki67 (n = 43)							
<30%	17	40	0	0	17	50	0.007
≥30%	26	60	9	100	17	50	
At diagnosis:							
Median age	57 (IQR 52-61)		59 (IQR 53-63)		56 (IQR 51-61)		0.036
MIPI (n = 147)							
LR	57	39	9	18	48	49	<0.001
IR	41	28	13	26	28	29	
HR	49	33	28	56	21	22	
Ki67 (n = 128)							
<30%	63	49	12	26	50	61	<0.001
≥30%	65	51	33	72	32	39	
Blastoid (n = 149)							
No	115	77	29	57	86	88	<0.001
Yes	34	23	22	43	12	12	
TP53 mutated (n = 94)							
No	69	73	12	40	57	89	<0.001
Yes	25	27	18	60	7	*11*	

ASCT = autologous stem cell transplantation, CNS = central nervous system, HR = high-risk, IR = intermediate-risk, Ki67 = cell proliferation marker, LR = low-risk, MIPI = mantle cell lymphoma international prognostic index, POD = progression of disease.

### Outcome after POD

With a median follow-up of 85 months for all 149 patients, the median OS from time of POD was 22 months (IQR = 9-73; Figure 1B). Obviously, OS decreased with subsequent PODs, that is, median OS was 11 months (IQR = 3-52), 8 months (IQR = 2-28), and 6 months (IQR = 2-16) after second, third, and fourth relapse, respectively (Figure 1B). Similarly, median PFS from start of relapse therapy decreased with subsequent lines of therapy; median PFS after second line was 8.6 months (IQR = 2.6-25), after third line 7.4 months (IQR = 1.3-16), and after fourth line 3.6 months (IQR = 1.4-7.3; Figure 1C). Finally, responses to therapy decreased over time with 96%, 67%, 43%, and 44% achieving complete or partial responses at first, second, third, and fourth line of therapy, respectively (Supplementary Figure 3, http://links.lww.com/HS/A121).

### POD before 24 months

**Figure 2.**
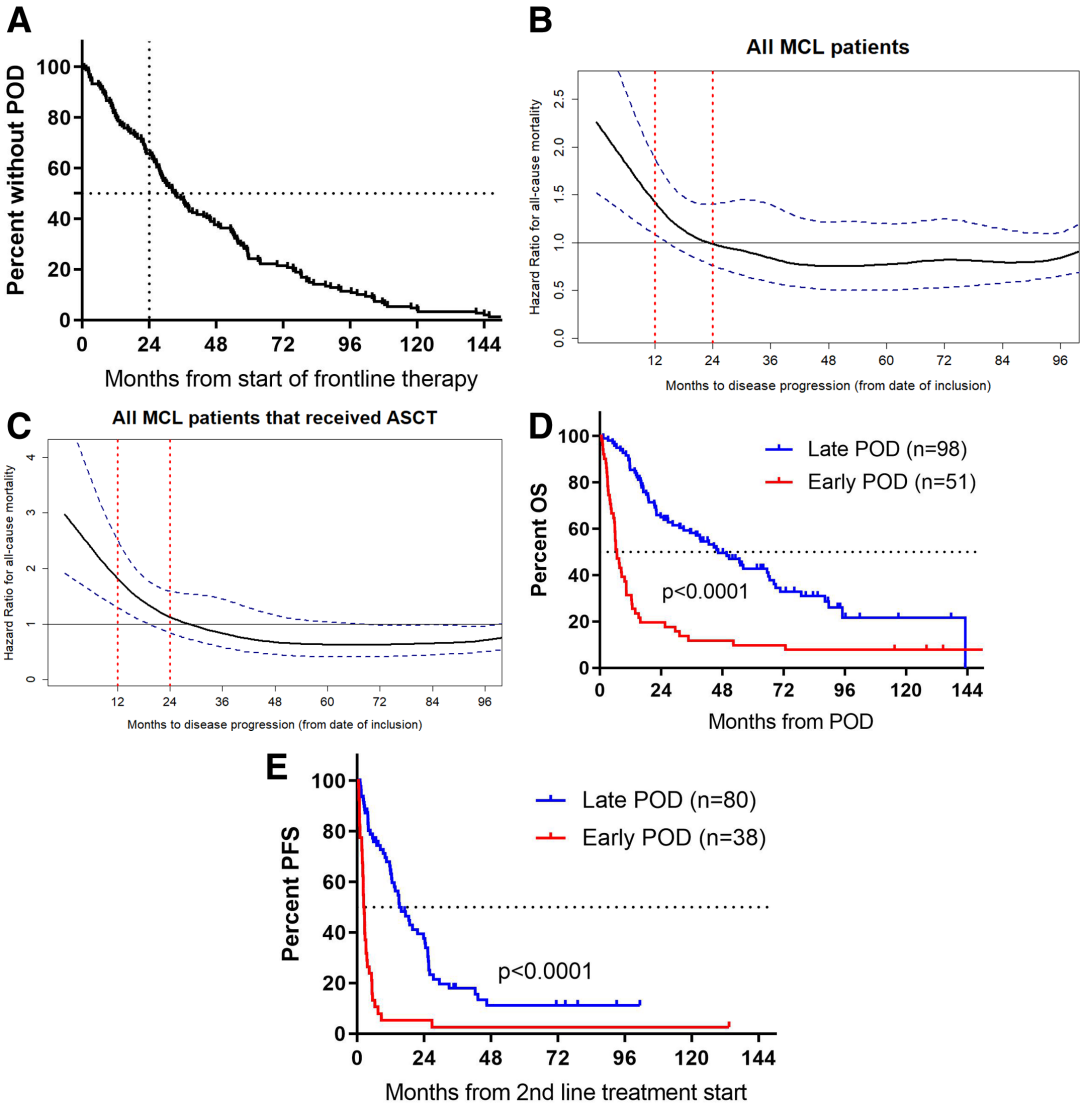
**Time to POD as a prognostic marker for outcome after POD.** (A), The time to first POD for all MCL2 and MCL3 with documented POD. (B), Spline curve showing the predicted hazard ratio of time to POD from start of frontline treatment as a prognostic marker. (C), Spline curve for patients who underwent ASCT. (D), OS and (E), PFS according to POD before or after 24 mo (POD24). ASCT = autologous stem cell transplantation, MCL = mantle cell lymphoma, OS = overall survival, PFS = progression-free survival, POD = progression of disease.

Visco et al^8^ recently showed that TTP was highly prognostic with a cut-off at 24 months (POD within 24 months, POD24). In our study, median TTP from frontline therapy was 33 months (IQR = 16-60, Figure 2A), and 24 months seemed a reasonable cut-off both by an intention-to-treat approach in all 149 patients (Figure 2B) and when focusing solely on the 126 patients who underwent ASCT (Figure 2C). The hazard ratio for death decreased rapidly with TTP during the first 24-36 months and since remained almost constant for later relapses. Median OS from first POD was 6.6 (IQR = 3.3-14) and 46 months (IQR = 18-95) in patients with early POD24 (n = 51, 34%) and later POD (n = 98, 66%), respectively (HR = 3.2 [95% CI = 3.0-7.8], *P* < 0.001, log-rank; Figure 2D). Ten patients progressed before ASCT and had a median OS of 5.3 months (IQR = 2.3-6.0; Supplementary Figure 4, http://links.lww.com/HS/A121), and 17 patients had POD later than 96 months and displayed a median OS of 55 months (IQR = 24-not reached).

For patients with available data on subsequent therapy, PFS from start of second line therapy was 2.3 (IQR = 1.6-4.3) and 15 months (IQR = 7-26) for patients with POD24 and later relapses, respectively (HR = 3.7 [95% CI = 2.1-6.4], *P* < 0.001, log-rank), and 2.2 months (IQR = 2.0-3.2) for primary refractory cases (Figure 2E, Supplementary Figure 5, http://links.lww.com/HS/A121). Patients who did not undergo ASCT for other reasons than POD (toxicity n = 7; harvest failure n = 5; patient choice n = 1) displayed a median OS and PFS of 40 and 19 months, respectively (Supplementary Figures 4, 5, http://links.lww.com/HS/A121).

Besides predicting for outcome and response to second line therapy, POD24 was significantly associated with inferior responses to all subsequent lines of therapy (Supplementary Figure 6, http://links.lww.com/HS/A121).

### Other prognostic markers

**Figure 3.**
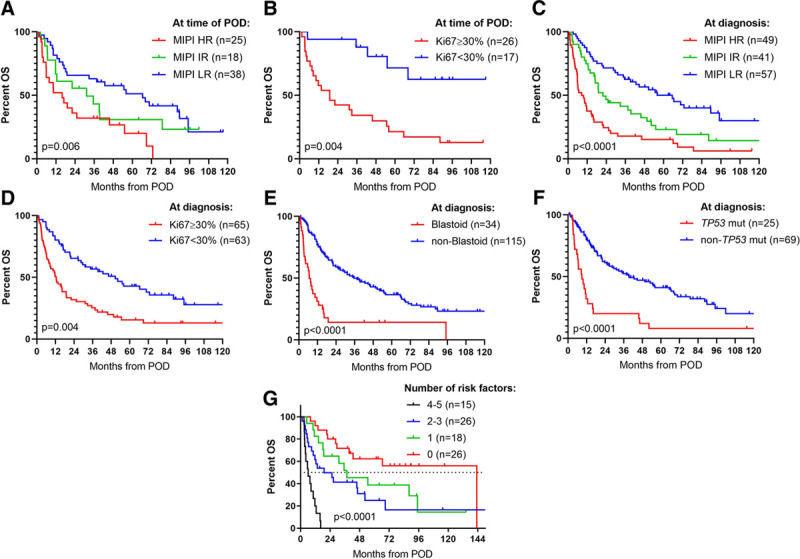
**OS after POD according to prognostic biomarkers.** (A) MIPI risk groups and (B) Ki67 expression measured at time of POD. (C) MIPI risk groups, (D) Ki67 expression, (E) blastoid morphology, and (F) *TP53* mutational status measures at diagnosis. (G) OS according to number of risk factor measured either at POD or diagnosis (POD24, MIPI high risk, Ki67 ≥30%, blastoid morphology, and *TP53* mutations). HR = high-risk, IR = intermediate-risk, Ki67 = cell proliferation marker, LR = low-risk, MIPI = mantle cell lymphoma international prognostic index, OS = overall survival, POD = progression of disease.

Both MIPI risk group (available for 82) and Ki67 ≥30% (available for 41) at time of POD showed prognostic value for OS (*P* = 0.006 and *P* = 0.004, respectively, log-rank; Table 2, Figure 3A, B). Despite the low numbers, both biomarkers showed independent prognostic value when adjusted for POD24 in multivariable Cox regression analyses (*P* = 0.0001 for MIPI and *P* = 0.11 for Ki67; Table 2). Due to missing data at time of POD, we investigated high-risk markers measured at time of diagnosis (MIPI, Ki67, blastoid morphology, and *TP53* mutations). In line with their obvious association with early POD, each marker held prognostic value for outcome after POD (Figure 3C–F). Interestingly, each diagnostic biomarker also retained independent prognostic impact after adjustment for POD24 (Table 2).

**Table 2:** Multivariable Cox Regression Analyses for OS After POD, Adjusted for POD24.

				OS		
	N	Adjustment	HR	95% CI, Lower	95% CI, Upper	*P*
At relapse						
MIPI	82	None	2.68	1.75	4.11	<0.0001
		POD24	2.36	1.53	3.65	0.0001
Ki67 >30%	41	None	3.66	1.46	9.16	0.006
		POD24	2.90	1.10	7.65	0.032
At diagnosis						
MIPI	147	None	2.03	1.63	2.54	<0.0001
		POD24	1.86	1.48	2.34	<0.0001
Ki67 >30%	128	None	2.25	1.48	3.42	0.0001
		POD24	1.87	1.22	2.87	0.004
Blastoid	149	None	3.21	2.07	4.98	<0.0001
		POD24	2.84	1.80	4.49	<0.0001
*TP53* mutations	92	None	2.81	1.67	4.73	0.0001
		POD24	1.81	0.97	3.40	0.064

CI = confidence interval, HR = hazard ratio, Ki67 = cell proliferation marker, MIPI = mantle cell lymphoma international prognostic index, OS = overall survival, POD24 = progression of disease before 24 mo from start of frontline treatment.

As an exploratory analysis, we combined the different biomarkers to perform a combined risk score for the prognosis after POD. Each high-risk marker was assigned 1 point: MIPI high-risk (either at diagnosis or relapse), Ki67 >30% (either at diagnosis or relapse), blastoid morphology at diagnosis, *TP53* mutation at diagnosis, and POD24. Despite the small numbers of each group, the analysis showed a clear separation of survival curves, and patients with 0, 1, 2-3, and 4-5 risk factors displayed 5-year OS of 62%, 39%, 31%, and 0%, respectively (Figure 3G).

### Comparison of relapse regimens

**Figure 4.**
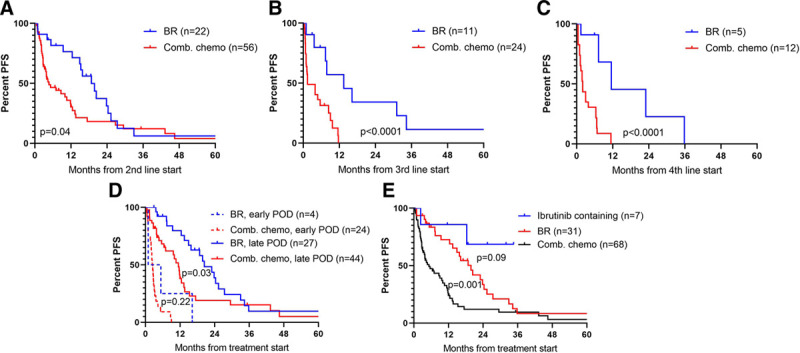
**PFS according to relapse treatment.** BR compared to other Comb. chemo regimens at (A) second line, (B) third line, (C) fourth line, and (D) displays BR vs Comb. chemo at all lines of therapy stratified by early and late POD (cut off 24 mo). (E) All lines of therapy compiled and stratified by BR, ibrutinib-containing, and other combination chemotherapy (In [D] and [E], each patient is only included once with the earliest line of treatment). BR = bendamustin-rituxumab, Comb. chemo = combination chemotherapy, PFS = progression-free survival, POD = progression of disease.

Relapse regimens were based mainly on chemotherapy and rituximab and are summarized in Supplementary Table 1, http://links.lww.com/HS/A121. We performed an exploratory comparison of the efficacy of the different combination chemotherapy regimens based on central agents as outlined in Supplementary Table 1, http://links.lww.com/HS/A121 (excluding novel targeted agents, low-dose/palliative regimens, monotherapy with rituximab or radiotherapy, and patients with central nervous system relapse; Supplementary Figure 7, http://links.lww.com/HS/A121). Bendamustine and rituximab (BR) was administered 38 times to 34 patients (5 times including also cytarabine [R-BAC], and once obinutuzumab instead of rituximab). Regardless of line of therapy, BR showed significantly prolonged PFS compared to all other combination regimens, most of which had higher toxicity profiles (Figure 4A–C, Supplementary Figure 7, http://links.lww.com/HS/A121). R-BAC did not seem to perform better than BR alone, and hence, they are evaluated combined (data not shown). In second line, overall response rate ([ORR] as defined by partial response [PR] and complete remission [CR]) of patients treated with BR (n = 21) was 86% (CR 57%) compared with an ORR of 54% (CR 30%) in patients treated with other rituximab-chemotherapy combinations regimens (*P* = 0.01, χ^2^). A similar trend was present in third line with ORRs of 60% (CR 60%) and 26% (CR 9%) for 10 and 23 patients, respectively (*P* = 0.063). In fourth line, ORR was 67% (CR 33%) and 46% (CR 18%) for 6 and 10 patients, respectively (*P* = 0.4). Importantly, administration of BR was also associated with a longer TTP from prior line of therapy, indicative of a lower risk disease. When adjusting for this in multivariable Cox regression analyses, BR no longer held positive predictive value for PFS. Only 4 patients with POD24 received BR (Figure 4D).

Due to the calendar year of therapy, only a minority of patients received novel drugs. Seven patients received ibrutinib, hereof 4 in the PHILEMON study (ClinicalTrials.gov, number NCT02460276; ibruntinib, lenalidomide, and rituximab).^12^ Compared with BR and other combination-regimens, the PFS for ibrutinib-containing regimens tended to be longer, despite being administered in later lines of therapy and with a shorter time from last line of therapy (Figure 4E).

Among patients with POD24, no single regimen showed superior response, and only 3 patients received ibrutinib. Similarly, for patients carrying other high-risk markers such as *TP53* mutations, no single regimen was associated with superior responses.

### Allogeneic stem cell transplantation

**Figure 5.**
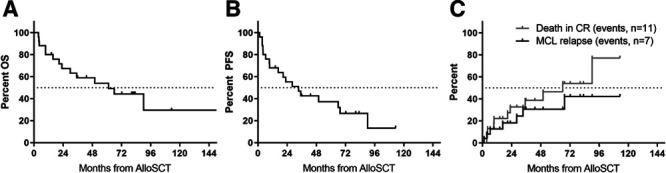
**Outcome after AlloSCT.** (A) OS, (B) progression-free survival, and (C) cumulative incidences of MCL relapses and MCL-unrelated deaths, respectively. AlloSCT = allogeneic stem cell transplantation, CR = complete remission, MCL = mantle cell lymphoma, OS = overall survival, PFS = progression-free survival.

Of the 149 relapsed patients, a total of 25 (17%) underwent AlloSCT. Median age at time of transplant was 56, and 17 were transplanted after second line therapy, 6 after third line, and 2 after fourth line. With a median follow-up of 82 months from time of AlloSCT, the median OS and PFS was 61.4 (IQR = 18-104) and 33.5 months (IQR = 9-90), respectively (Figure 5A, B). After 2 years, 18% of patients had experienced MCL relapses, whereas 29% had died from MCL unrelated reasons mainly associated with treatment-related toxicity (Figure 5C). Among the AlloSCT patients, the presence of high-risk markers was significantly lower than in nontransplanted patients. Only 3 patients with POD24 underwent AlloSCT, and they all displayed rapid progression after transplantation (PFS 1, 4, and 9 months, respectively); however, one of these patients since achieved a long-term remission after local radiotherapy. These 3 patients all had *TP53* mutations at diagnosis, compared to none of 15 other AlloSCT patients with available diagnostic sample for mutational analysis.

## Discussion

We present the results of postrelapse outcomes of patients included in the 2 prospective Nordic MCL trials, MCL2 and MCL3, for patients younger than 66 years of age. We confirm the impact of POD24 as a prognostic and predictive marker, and we show that well-known prognostic markers measured both at diagnosis and at time of relapse retain prognostic value independently of POD24.

Stratifying patients at relapse may be used to allocate patients of different risk profiles to different salvage regimens. Especially, to choose between repetition of chemoimmunotherapy or attempting novel targeted agents, and subsequently, if the patient should proceed to an allogeneic transplantation. Due to the calendar year of most treatments in this study, most patients were not treated with novel targeted agents. However, it represents a long-term follow-up of the prospective cohort of patients treated by standard of care frontline regimens.

We confirmed the results by Visco et al^8^ that the POD24 could be used as a prognostic discriminator of patients who were treated by intensive frontline regimens. Patients with POD24 displayed a median OS of 6.6 months compared with 46 months for patients with later relapses. Similarly, treatment responses were highly different between the 2 groups, not only at second line but also subsequent lines of therapy.

By comparison of all combination chemotherapy regimens used in the relapse setting, BR performed better than all others despite a lower toxicity profile. However, administration of BR was also associated with longer remission after last line of therapy, and hence, treatment groups were not comparable. Adjusting for response duration to last line of therapy, BR was no longer significantly better than other salvage regimens. Nonetheless, our results support the continued use of BR in patients with longer preceding remissions. Recently, BR alternating with high-dose cytarabine followed by ASCT also proved to be well tolerated and achieving high rates of durable remission in first line.^13^

Patients with POD24 displayed dismal outcomes and showed very poor response rates to all subsequent regimens. Hence, novel treatment approaches are warranted for this subset of patients. Ibrutinib has shown impressive overall responses in R/R MCL in general.^14^ However, still for ibrutinib, TTP impacts the responses and response durations markedly. Thus, this may not be sufficient as a single agent for patients with early relapses.^9^

POD24 was not surprisingly associated with other known prognostic markers such as MIPI, Ki67, blastoid disease, and *TP53* mutations. Despite this, each of these biomarkers also showed independent impact irrespective of POD24, and furthermore, this was true whether measured at initial diagnosis or at the time of POD. This illustrates the heterogeneity of MCL, that prognostic stratification from diagnosis will continue also after POD. In an exploratory analysis, we combined the different risk markers to perform a risk score for post-POD survival. Most importantly, we defined a low-risk group of which 62% were alive 5 years after first POD. This analysis was affected by missing data and small risk groups, and hence, it does not represent a model for future use. However, it shows the complexity and impact of many different prognostic biomarkers and supports a detailed evaluation of each patient to assess their risk profile. It may identify patients with no high-risk markers and a very good prognosis, and other patients with many high-risk markers who will most likely not respond to conventional chemoimmunotherapy.

A total of 25 patients from our cohort underwent AlloSCT. With a median PFS of 33.5 months from time of transplantation and no clear survival plateau, the overall results were not supportive of AlloSCT. This may be due to the relatively high rate of nonrelapse mortality, which has also been observed in other studies of AlloSCT in MCL.^15^ AlloSCT was of particular interest in high-risk groups such as patients with early POD, since no other therapy has shown convincing results for this subset. However, only 3 patients with POD24 went on to AlloSCT and they displayed variable responses. The underrepresentation of early progressors may be due to the lack of durable responses needed to bridge the patients to transplantation.

One of the strongest prognosticators for MCL is mutations of *TP53*.^11,16^ Lin et al^17^ suggested a role of AlloSCT for these patients; however, in our study, only 3 patients with *TP53* mutations (all early POD) underwent AlloSCT, and all progressed again within 1 year of transplant. Prospective data are warranted to evaluate the efficacy of AlloSCT in *TP53* mutated patients. Importantly, Wang et al^18^ recently published that chimeric antigen receptor T-cell therapy was efficacious in relapsed MCL regardless of high-risk biomarkers; although they did not include TTP as a biomarker. This supports the use of such treatment already at first POD in high-risk patients, or for some maybe already at frontline. Even though ibrutinib may not overcome the very high-risk MCLs such as early relapses and *TP53* mutated, it may serve as bridging strategy to therapies such as AlloSCT and chimeric antigen receptor T-cell (CAR-T).^9,19^

In this study, we mainly investigate the use of chemoimmunotherapies, and the results overall support the continued use of, for example, BR in patients with few risk factors present at relapse. However, as mentioned, there is an unmet need in patients with early POD or other high-risk markers. With many novel targeted therapies appearing, studies evaluating these in high-risk R/R MCL patients will be of high interest. Accordingly, the few cases of these novel therapies is an important limitation to the current study. Furthermore, the retrospective nature of the study reduces the ability to compare different regimens across subgroups of patients. However, the predefined study cohort, that is, participation in the Nordic MCL2 and MCL3 trials, increase the comparative.

In conclusion, this report displays the postrelapse outcomes of patients originally treated in the 2 Nordic MCL frontline trials consisting of intensive cytarabine-containing induction therapy and ASCT. We confirm the prognostic impact of the POD24 which has been suggested by others, and we highlight the relevance of other known prognostic biomarkers, whether measured at diagnosis or re-evaluated at time of POD. Patients with early POD or other high-risk markers showed very poor responses and outcomes in general, and further studies exploring novel treatment modalities are needed to guide handling of this subset.

## Acknowledgments

The authors thank the medical and nursing staffs of all contributing departments to this study, and the patients for their willingness to participate.

## Sources of funding

This study was supported by grants from the Independent Research Fund Denmark, the Novo Nordisk foundation, Rigshospitalet’s Research Foundation, Lundbeck Foundation, Danish Cancer Research Foundation. Furthermore, the K.G. lab is funded by center grants from the Danish Cancer Society (Danish Research Center for Precision Medicine in Blood Cancer; grant 223-A13071-18-S68), the Novo Nordisk Foundation (Novo Nordisk Foundation Center for Stem Cell Biology, DanStem; grant NNF17CC0027852), and the Greater Copenhagen Health Science Partners (Clinical Academic Group in Translational Hematology).

## Disclosures

IG has received IG Honoraria from Janssen. CUN has received consultancy fees and/or travel grants from Janssen, Abbvie, Novartis, Roche, Sunesis, Gilead, AstraZeneca, and CSL Behring, and research funding from Novo Nordisk Foundation, grant NNF16OC0019302, Abbvie, AstraZeneca and Janssen, outside this project. All the other authors have no conflicts of interest to disclose.

## Supplementary Material


