# Supplementary material for: Detailed Long-Term Follow-Up of Patients Who Relapsed After the Nordic Mantle Cell Lymphoma Trials: MCL2 and MCL3
Source: Hemasphere. 2020 Dec 21;5(1):e510. doi: 10.1097/HS9.0000000000000510 (PMC7755521; doi:10.1097/HS9.0000000000000510)
Supplement: Supplementary file 1 [file hs9-5-e510-s001.docx]

**Potent preclinical efficacy of DuoHexaBody-CD37 in patients with newly diagnosed and relapsed/refractory B-cell malignancies**

**Supplemental Material and Methods**

**Therapeutic Antibodies**

DuoHexaBody-CD37 and the negative control anti-HIV-1 gp120 antibody IgG1-b12 (mentioned in manuscript as IgG1-ctrl) were generated by Genmab (Utrecht, The Netherlands) as previously described (S.C. Oostindie et al., 2019). Rituximab (MabThera®) and ofatumumab (Arzerra®) were obtained from Genmab.

**Patient samples**

According to the Dutch Central Committee on Research involving Human Subjects (CCMO), this type of study does not require approval from an ethics committee. Primary patient samples wereh collected at Amsterdam University Medical Center, location VUmc, according to the *code of conduct for medical research*. Tissues from lymph node biopsies were mechanically disrupted, cultured overnight in a fully humidified incubator and the cells in suspension were collected and cryopreserved in liquid nitrogen until further use. For 7 CLL samples and 1 MCL sample tumor cells were isolated from mononuclear cells of the bone marrow (BMNCs) or peripheral blood (PBMCs) by density-gradient centrifugation (Ficoll-Paque PLUS, Cytiva, MA, USA) according to manufacturer’s instructions, and were either used directly or cryopreserved in liquid nitrogen until further use.

**Tumor-specific surface markers**

B cells were identified using flow cytometry by gating for CD3^-^CD19^+^ cells within the CD45^+^ population and made up 20-80% of the total lymph node cell population. Malignant B cells were identified by kappa/lambda staining to screen for clonality and if possible also by the tumor-specific markers CD10^+^ (FL and DLBCL), CD5^+^ (CLL), CD5^+^CD23^-^ (MCL). The following antibodies were used: CD45-KO and CD19-PC7 (Beckman Coulter, CA, USA), CD3-V450, CD5-APC, CD10-APC-H7 and kappa-APC-H7 (BD, NJ, USA), CD5-PE, CD10-PE and kappa-PE (DAKO, Agilent Technologies, CA, USA), CD23-FITC (BioLegend, CA, USA) and lambda-FITC (Emelca Bioscience, Clinge, Netherlands).

**Quantitated expression assays**

Target cells were incubated with purified mouse anti-human IgG1 antibodies against CD37, CD46, CD55, CD59 and CD20 (BioLegend) and expression levels were quantified by flow cytometry using an indirect immunofluorescence assay (QIFIKIT®, Agilent Technologies) according to manufacturers protocol, followed by a staining for tumor specific cell surface markers.

**Complement-dependent cytotoxicity assays**

Complement-dependent cytotoxicity assays were performed by incubation of single cell suspensions (1x10^5^ cells/well) with serial dilutions (0.01-10 µg/mL) of DuoHexaBody-CD37 for 45 minutes at 37°C in the presence of 20% normal human serum (NHS; pooled from 8 healthy donors) as a source of complement. Cells were analysed by flow cytometry and viability (%) and lysis (%) of malignant B cells was determined according to the following formulas:

$$\%viability=100*\left( \frac{\%7AAD-negative cells of test sample}{\%7AAD-negative cells of control sample} \right)$$

$$\%lysis=(100-\%viability)$$

**C1q binding assays**

Target cells were incubated with DuoHexaBody-CD37 (10 µg/mL) and purified human complement component C1q (2.5 µg/mL; Quidel, San Diego, CA) for 45 min at 37°C. After washing, purified C1q was labeled with a fluorescein isothiocyanate (FITC)-conjugated rabbit anti-human C1q antibody (Dako, Glostrup, Denmark). Binding was detected by flow cytometry and expressed as the median fluorescent intensity (MFI). MFI values of purified C1q labeled with FITC anti-human C1q were subtracted by MFI values of negative control (FITC-anti-human C1q only).

**Statistical analysis**

Flow cytometry data were analyzed using FACS DIVA software. Graphs were plotted using GraphPad Prism 8.2. for Windows. Statistical analysis was performed with the appropriate parametric or non-parametric test, as indicated in the figure legends. Two-sided P values < .05 were considered statistically significant.

**Supplemental Figures**

**
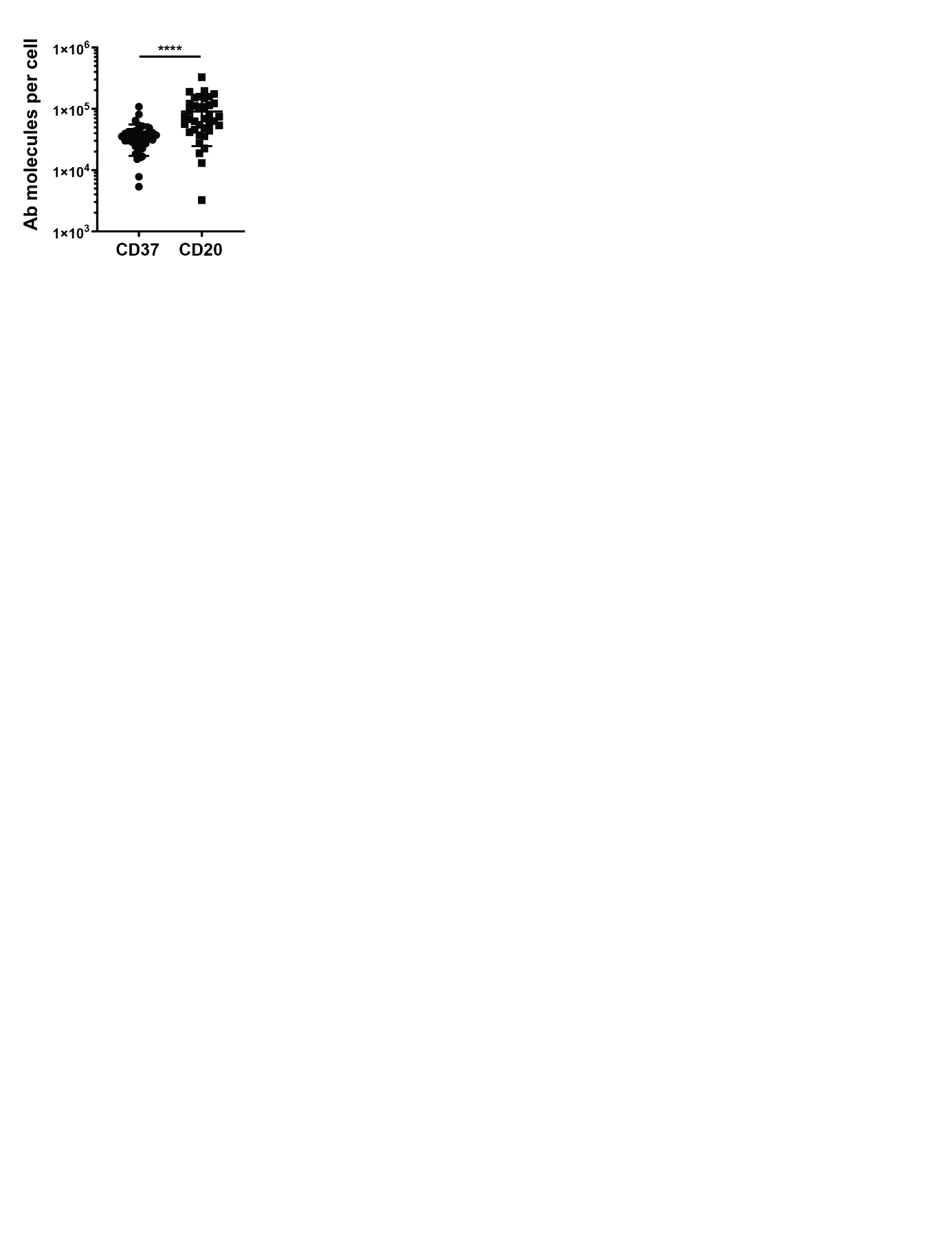
Supplemental Figure 1.** Expression levels (antibody molecules per cell) of CD37 and CD20 on tumor B cells in samples from ND patients (****p<0.0001; Wilcoxon matched-pairs signed rank test). Data are shown as the median and interquartile range.

**
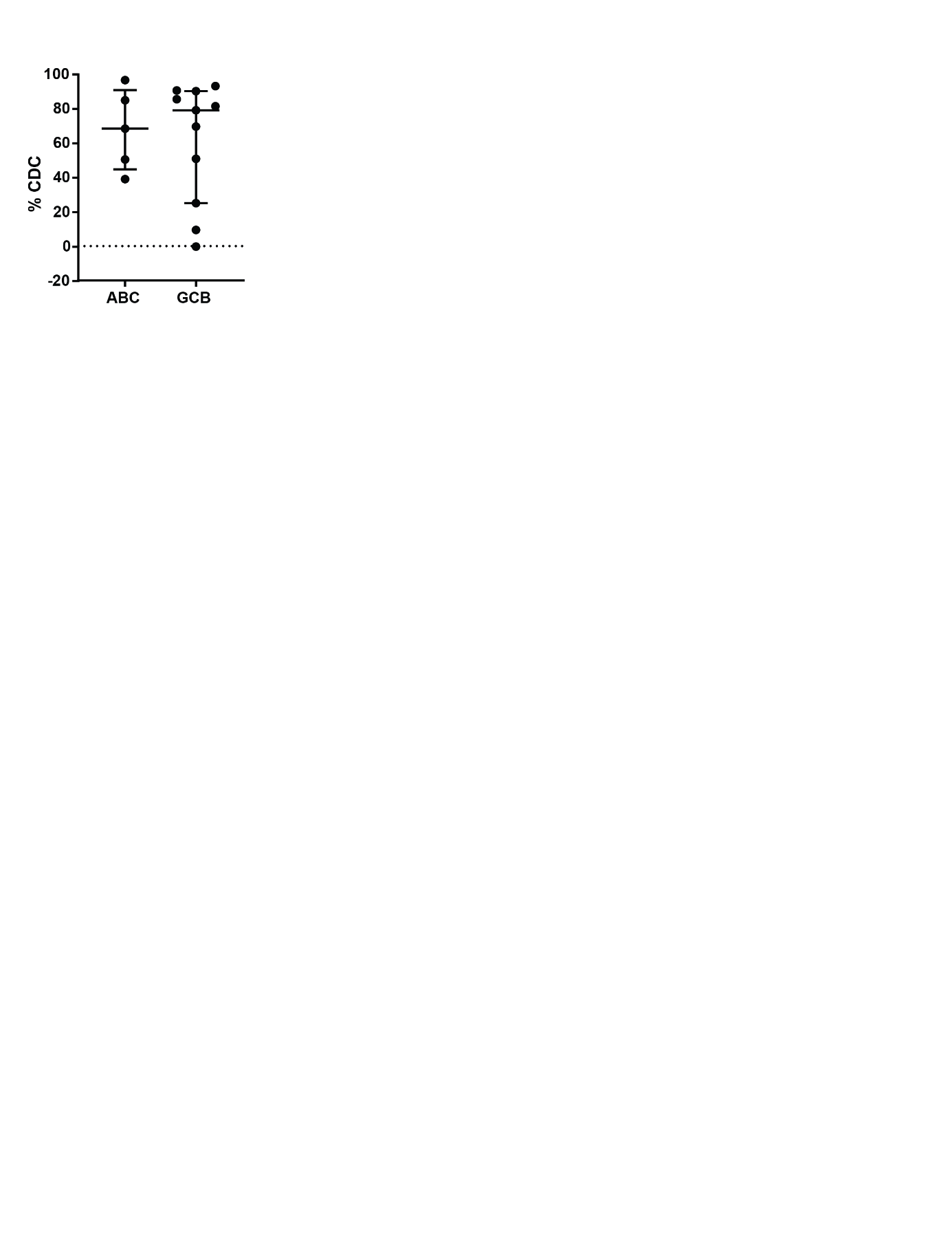
**

**Supplemental Figure 2.** CDC activity of DuoHexaBody-CD37 (10 μg/mL) in DLBCL patient samples of ABC (n=5) and GCB subtype (n=11) (ns; Mann-Whitney U-test). Data are shown as the median and interquartile range.

**
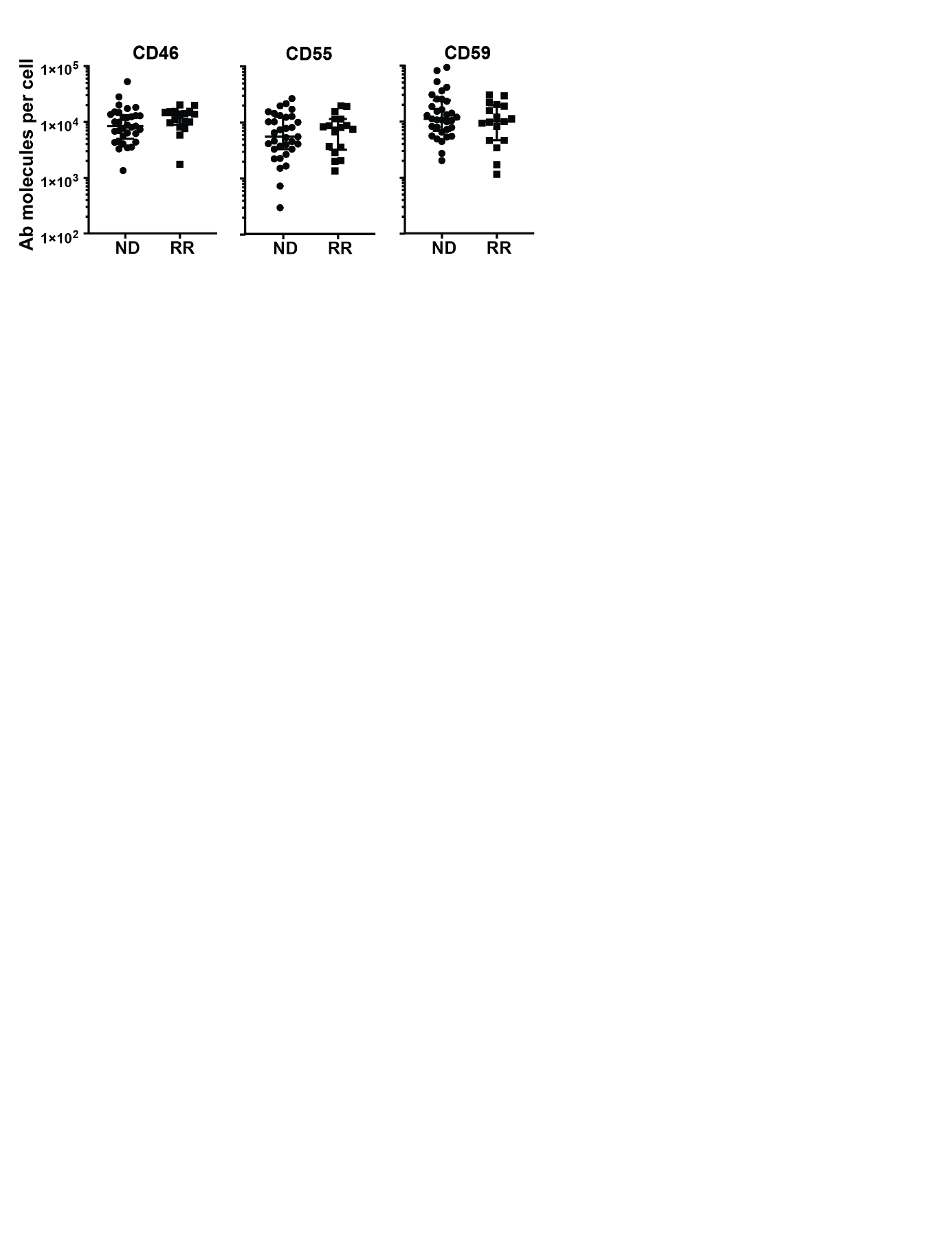
**

**Supplemental Figure 3.** Expression levels (antibody molecules per cell) of complement regulatory proteins CD46, CD55 and CD59 on tumor B cells, according to treatment status; ND versus RR (not significant; Mann-Whitney U test). Data are shown as the median and interquartile range.


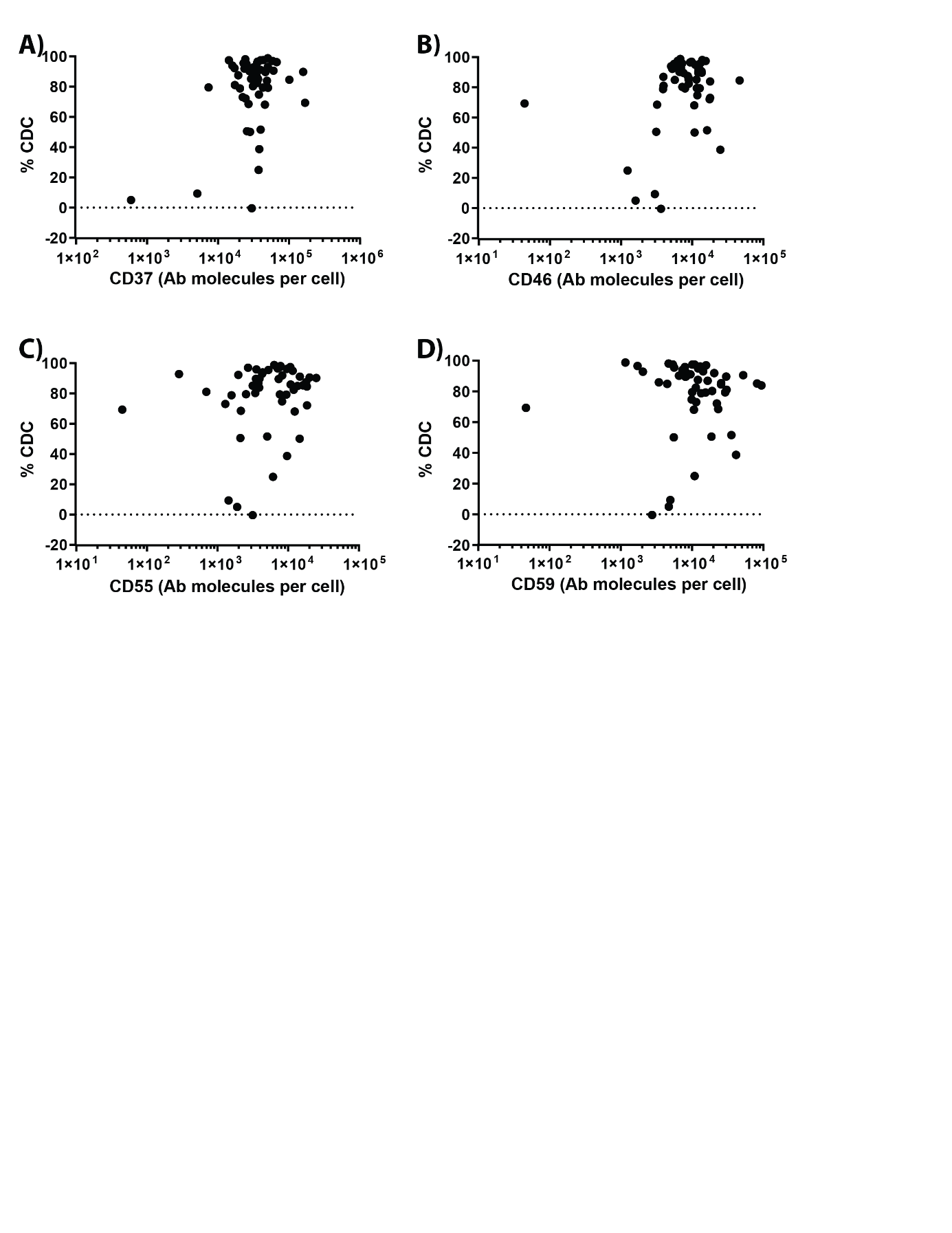


**Supplemental Figure 4.** CDC activity of DuoHexaBody-CD37 (10 μg/mL) correlated with quantified expression levels (antibody molecules per cell) of **(A)** CD37 and **(B-D)** complement regulatory proteins CD46, CD55 and CD59 (Spearman’s correlation r=0.1408, p=0.3243; r=0.1929, p=0.1751; r=0.1901, p=0.1814; r=-0.2024, p=0.1542, respectively). Data are shown relative to a no antibody control sample.


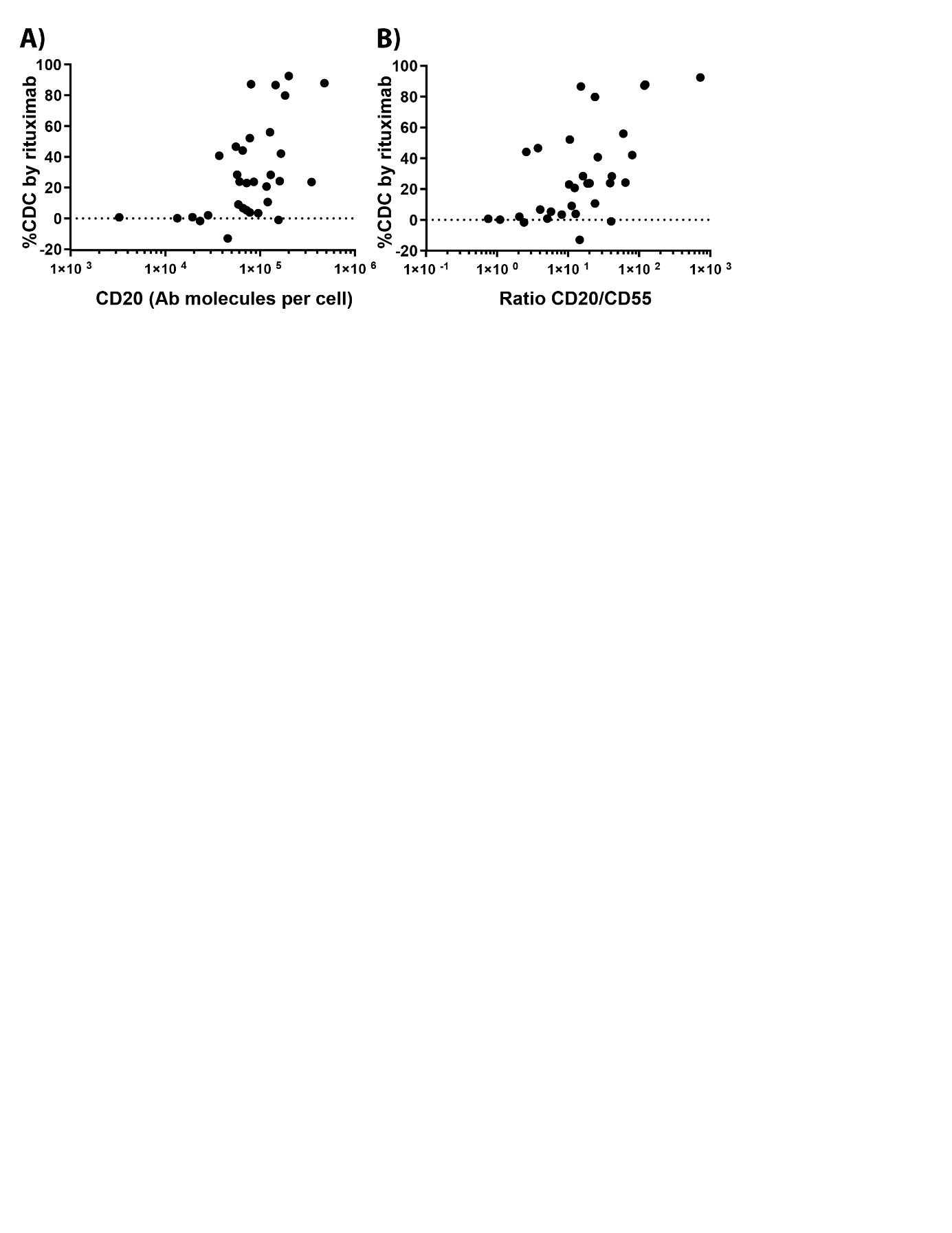


**Supplemental Figure 5.** CDC activity of rituximab (10µg/mL) correlated with **(A)** quantified CD20 expression (antibody molecules per cell; Spearman’s correlation r=0.5488, **p=0.0011) and **(B)** the ratio of expression levels of CD20 to complement regulatory protein CD55 (Spearman’s correlation r=0.5957, ***p=0.0003). Data are shown relative to a no antibody control sample.
